# Reducing Detrimental Communication Failure Impacts in Microgrids by Using Deep Learning Techniques

**DOI:** 10.3390/s22166006

**Published:** 2022-08-11

**Authors:** Babak Arbab-Zavar, Suleiman M. Sharkh, Emilio J. Palacios-Garcia, Juan C. Vasquez, Josep M. Guerrero

**Affiliations:** 1 AAU Energy, Aalborg University, DK-9220 Aalborg, Denmark; 2 Faculty of Engineering and the Physical Sciences, University of Southampton, Southampton SO17 1BJ, UK; 3 Department of Electrical Engineering (ESAT), KU Leuven, ELECTA, BE-3001 Leuven, Belgium; 4 EnergyVille, Thor Park 8310, BE-3600 Genk, Belgium

**Keywords:** microgrid, machine-to-machine communication, deep learning, time series forecasting, artificial neural networks

## Abstract

A Microgrid (MG), like any other smart and interoperable power system, requires device-to-device (D2D) communication structures in order to function effectively. This communication system, however, is not immune to intentional or unintentional failures. This paper discusses the effects of communication link failures on MG control and management and proposes solutions based on enhancing message content to mitigate their detritus impact. In order to achieve this goal, generation and consumption forecasting using deep learning (DL) methods at the next time steps is used. The architecture of an energy management system (EMS) and an energy storage system (ESS) that are able to operate in coordination is introduced and evaluated by simulation tests, which show promising results and illustrate the efficacy of the proposed methods. It is important to mention that, in this paper, three dissimilar topics namely MG control/management, DL-based forecasting, and D2D communication architectures are employed and this combination is proven to be capable of achieving the aforesaid objective.

## 1. Introduction

The idea of localizing energy generation and consumption is the backbone of microgrid (MG) philosophy. In case of grid-connected MGs, the localization concept can be beneficial for both urban and rural power-systems by reducing energy exchange with the main power-lines while utilizing more renewable energy resources. Minimizing power exchange with the grid during peaks demand or supply results in important benefits [1]:

- Preventing possible and harmful over-voltage problems especially in weak-grids;
- Avoiding over-designed power-lines due to internal power balancing of the units;
- Improving grid’s power quality and contributing to better grid stability.

However, due to the inevitable occurrence of internal energy imbalances caused by generation and consumption mismatches, power exchanges with the grid at the point of common coupling (PCC) is unavoidable. Therefore, in most MG architectures energy storage systems (ESS) are included to compensate for power imbalance. These ESSs are controlled by energy management systems (EMS) that regulate the power flow optimally. To achieve a valid operation of such a power system, communication links between these different energy players are required. These links transmit power measurements from different generation/consumption stations and the ESS charge status to the EMS where the calculations are conducted and proper power references for the ESS are set and transmitted [2]. The system’s normal functionality is hindered when these links malfunction.

As an idealistic prospect, the future power network can be formed by clusters of independent MGs that are able to individually localize their generation and consumption by design. These MG units will be able to interoperate and exchange energy when excess in one MG can balance a deficit in another. This, inevitably, requires energy awareness between neighboring units through communication links. In other words, the role of communication is not limited to be within an MG and between the individual devices but it also plays a much wider role [3,4].

In an MG structure, if the communication links fail due to either unintentional reasons such as natural disasters, or intentional purposes such as cyber attacks, the effects are not just limited to poor power regulation. In fact, it can contribute to severe problems such as MG bus voltage and frequency deviation from their reference values or milder effects such as losing the optimal energy balance of the system. In any case, normal operation is no longer possible without an operational communication infrastructure [5].

The communication link loss resulting complications can be mitigated if control structures of power converters that interface the units (for instance an ESS) with the MG buses are able to estimate their operational set-points for the next time steps. Specifically in this case, if consumption and generation forecasting is available for a certain time horizon the future ESS power reference can be calculated by the EMS and transmitted to its controller in advance. These redundant information will be stored on a memory buffer at the controller side and be utilized in case of a communication link loss occurs. This criteria is relatively simple if the generation or consumption follow a known and predictable pattern. For example, in the vehicle-to-grid (V2G) system described in [6] the generation and load are based on an electric train’s acceleration and breaking due to arrivals and departures which are precisely scheduled. By contrast, in the type of power-systems that we are interested in this paper, such as residential MGs, it is a quite different story: forecasting the generation that is based on renewable energy supply, which is volatile in nature, or the consumption, that can largely vary based on the users’ decisions, is not a trivial problem. However, there are techniques that have proved that even in these circumstances the generation and consumption can be forecasted for a short-term time horizons (one hour with time steps in terms of minutes). These forecasts are achieved based on utilizing deep learning (DL) methods [1,7,8,9,10,11,12]. These related contributions mainly focus on the forecasting aspect while in this paper we attempt to utilize such prediction methods to enhance and rearrange the message content from the communication point of view while investigating the potential challenges that are accompanying this approach.

In this paper, since some dissimilar, yet, related topics such as MG control, device-to-device (D2D) communication, and DL based forecasting techniques are combined different approaches were utilized for validation and analysis to achieve the desirable proof of concept. In this regard, link reliability verification and latency analysis were also conducted to show the capabilities and also limitation of the transmission system. On the other hand, short-term and very short-term time-series forecasting was utilized to demonstrate how to predict future time-steps for generation and production. Most importantly, a grid-connected MG model based on the operation of grid-feeding inverters was utilized as a base for the simulation platform so that the aforementioned transmission architecture and the forecasting system can be integrated and the required tests can be conducted.

The main contributions of this paper are listed as follows:

- The detrimental communication link failure effects on the internal energy balancing of MG systems are investigated. The specific MG examined in this study is based on grid-feeding inverters and operates in grid-connected mode. In this configuration, the communication link transmits measurements from the consumption, production, and storage stations to the EMS. Furthermore, it relays power references from the EMS to the ESS so that the storage system can effectively contribute to minimizing the PCC power exchange.
- Methodology of short-term load and PV-generation forecastings based on DL methods are described and numerically performed on sample datasets for a residential MG application.
- The details of the designed communication infrastructure are explained and evaluated in terms of the dependency of latency values to payload size.
- A system architecture that can enhance communication message content is proposed. The effectiveness of this system is evaluated by comparing the PCC power exchange measurements while the communication link is operational using forecasted values following communication link failure.

The remainder of the paper is organized as follows. Section 2 introduces the role of communication in MG management. The effect of communication link loss on MG performance is introduced and discussed in Section 3. Section 4 presents DL based methods for time-series forecasting. The description of the selected communication system is provided in Section 5. Section 6 is dedicated to describe the proposed system architecture for enhancing the communication message content. Finally, conclusion and discussions are presented in Section 7.

## 2. Communication System for Residential MG Management

**Figure 1:**
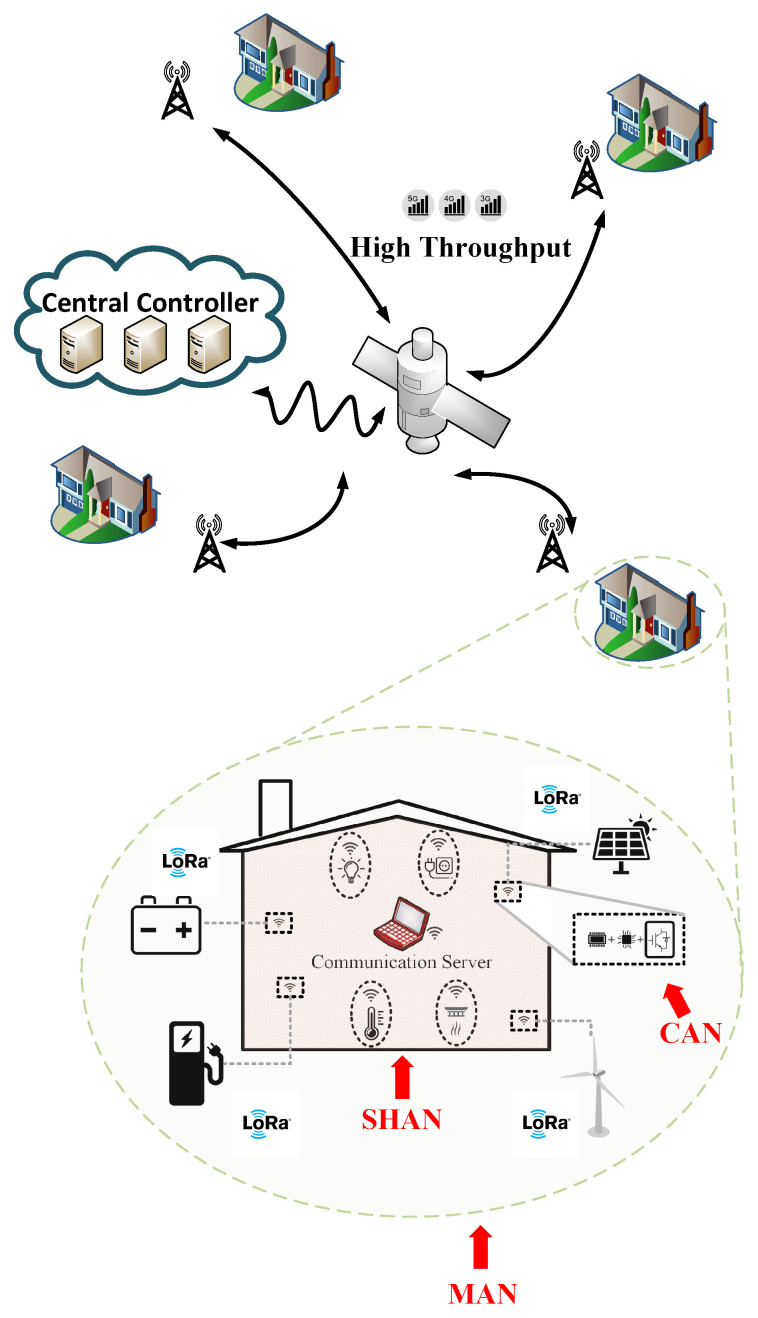
Multilevel communication network for a system of residential MGs for remote applications.

Figure 1 is a conceptual illustration of a combination of different communication systems operating in coordination to manage an MG cluster-based power-system. It consists of several modern farmhouses that can communicate through a satellite type backhaul link due to the wide separation and possible terrain complications between them. They are also electrically inter-connected which is not shown in the figure. By zooming on to an individual farmhouse, which represents a residential MG, it can be understood that distributed-generators (DGs) and loads are required to be managed by central controllers that can be located relatively far away. That long-range requirement can justify the utilization of LoRa, which is a low-power and long-range wireless protocol [13].

Zooming in further, the core of the residential MG can be considered as a building that should be equipped with device-to-device (D2D) communication capabilities to supervise indoor and outdoor smart-devices. In this case, wide coverage is no longer required and ease of implementation and scalability are the important required characteristics that define the selected communication protocol. ZigBee is a very good candidate for smart-homes [14,15]. In line with the development of MG, their low level control structures became less communication dependent and more reliant on local measurements. However, communication is still required for the higher level of the MG control hierarchy and only the primary level operates as communication free [16,17,18].

The lowest level of communication, consists of power-electronics-based converters that interface the DGs and storage systems to the buses. These devices require access to critical readings (namely voltage and current measurements from sensors) in order to regulate their output characteristics based on their embedded control algorithms. This accessibility requires other types of communication links which can be very different from the higher levels in terms of specifications.

From a communication network point of view, the illustrated setting consists of four levels of networks that are briefly summarized in Table 1. Those levels are device area network (DAN), smart home area network (SHAN), microgrid area network (MAN), and finally multimicrogrid area network (MMAN). As it can be observed, the specifications/requirements and protocols of each of the levels differ considerably from each other.

## 3. Link Loss Effects

Based on the descriptions of the multilayer communication system provided in Section 2 it can be understood that link failures can happen at each of the levels with different consequences. For example, at the device level, where the acceptable level for latencies are very small (in the order of 10 milliseconds or less) even small delays can cause stability problems and be very harmful. In contrast, at multimicrogrid level, a total link failure may cause serious economic problems or voltage and frequency deviations.

**Figure 2:**
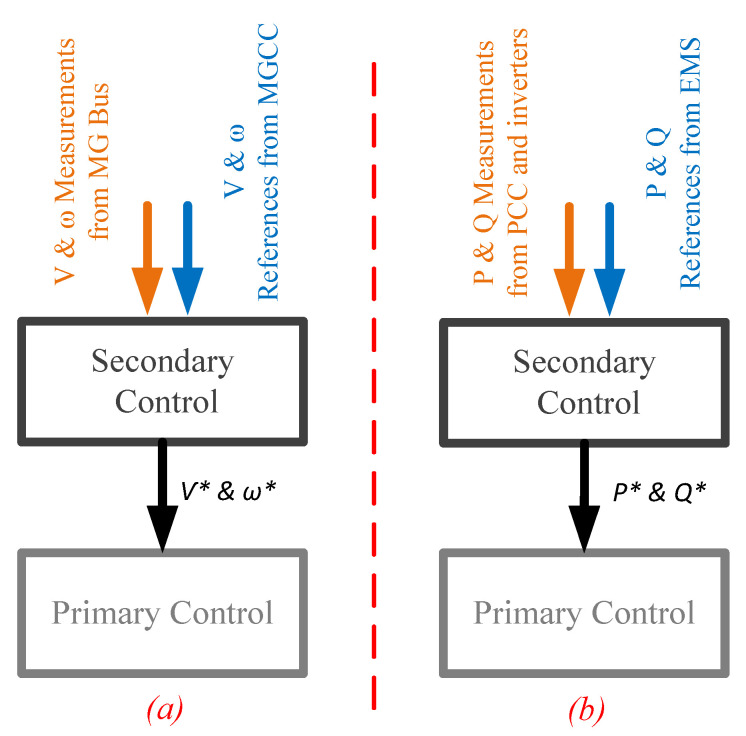
Secondary control for different types of inverters. (**a**) Grid-forming and VSI. (**b**) Grid-feeding and CSI. The values with a star superscript represent secondary control references.

In this paper, we are primarily interested in the MG-level and the details of its link failure effects will be discussed in this section. Based on the configuration of a specific MG the role of communication in the control architecture varies. For example, in an MG architecture based on voltage source inverters (VSIs) the secondary control is the most recognizable point of communication interaction with MG control. This level deals with voltage and frequency deviations caused by the primary control by restoring the reference values. Furthermore, it contributes to proper reactive power sharing since there is a trade-off between voltage regulation and reactive power sharing in the primary level [29,30]. In another grid-connected MG topology, that is formed of grid-feeding inverters, the secondary control functionality is completely different. Figure 2 illustrates the difference between the secondary-level of control for the two aforementioned types of inverter. As it can be observed, when voltage-source and VSIs are used the secondary control inputs are voltage/frequency references and measurements while in the grid-feeding and current source inverters (CSIs) externally generated power references and power measurements are transmitted to the secondary controller. As a result of this argument it can be easily understood that the effects of communication link losses can be quite different depending on which types of inverters are utilized.

### 3.1. Impact of Link Failure on a Grid-Connected MG with Grid-Feeding Inverters

In order to demonstrate the effects of communication link loss on the power regulation of a typical residential MG an experimental testbed was prepared and a test scenario was created. The rationale behind this test was to show how an external EMS that transmits power setpoints for the ESS, through the communication network, can contribute to a more desirable MG internal power balancing and minimize the PCC power exchange.

In the setup, three grid-feeding 2.2 kW *danfoss* inverters were utilized with a shared DC link and an AC grid-connection point of common coupling (PCC). These inverters represent *consumption, PV production, and an ESS* that is considered fully charged at the beginning of the day. In this experimental simulation consumption and PV generation profiles were synthetically generated (based on real data) at 1-minute resolution for a one-day time horizon.

**Figure 3:**
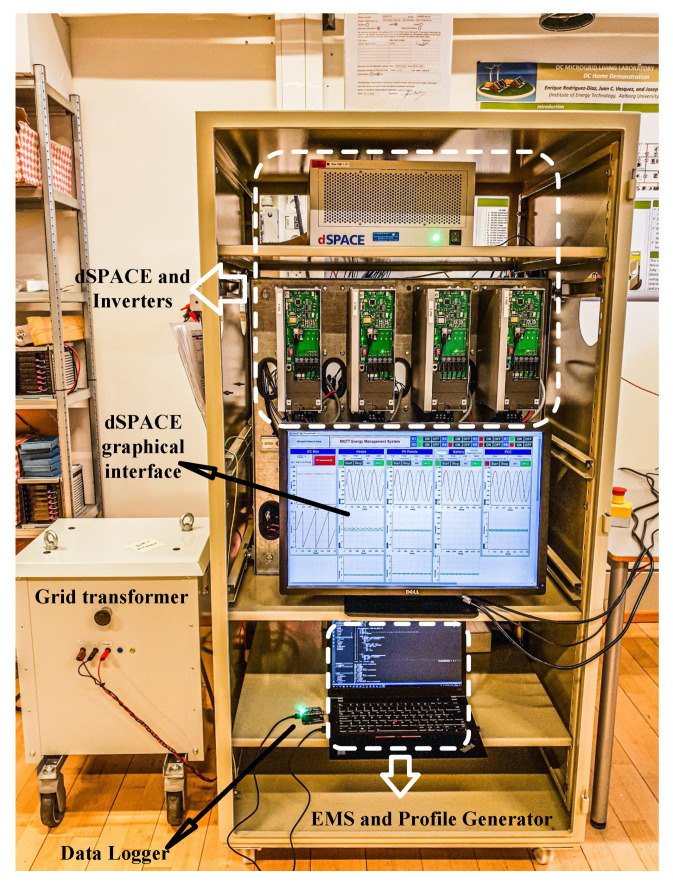
Experimental setup.

An external EMS that receives the power measurements from the inverters and calculates and transmits proper power references for the ESS is included in our testbed. This EMS is connected to the other devices through a message queuing telemetry transport (MQTT) communication structure that was created by using micro-python enabled microcontrollers [2]. A photo of this experimental tetstbed is provided in Figure 3.

**Figure 4:**
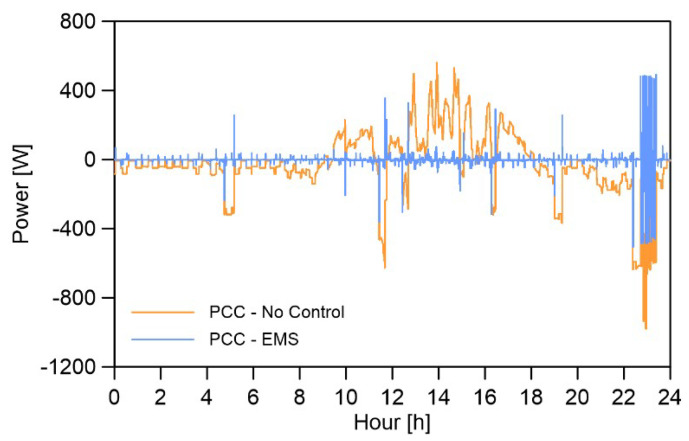
PCC test results. Positive and negative signs indicate the direction of PCC power exchange, import to the MG, and export from the MG, respectively.

The PCC power and energy exchange results are numerically presented by comparing the normal scenario (with an operational communication system) with a no control scenario that emulates communication link loss. The results are illustrated in Figure 4. A comprehensive descriptions of the experimental setup including the communication infrastructure and the MG control algorithms are provided in a previously published research article by the authors [2].

Table 2 summarizes the results, the first row represents 24 h energy values while the power exchange root mean square error (RMSE) indicators are provided in the second row. These RMSEs are calculated assuming that the ideal situation is zero PCC power exchange.

As it can be observed the PV production (2.145 kWh) is not enough to meet the demand (−2.599 kWh) in the 24 h of the test period. In other words, internal energy balancing cannot be achieved without energy exchange with the stiff-grid or utilizing an operational ESS. In the ESS model used for this experiment [2], the initial state-of-charge (SoC) level was assumed to be one so that it can compensate for the energy deficit effectively.

The forth and fifth columns of the table present the PCC energy exchange and power RMSE results for two scenarios of communication link down (PCC no control) and operational communication system that transmit ESS power references generated by the external EMS (PCC EMS). As it can be observed, if the communication link fails the daily PCC energy exchange that is (0.019 kWh) exported to the grid will be changed to (−0.455 kWh) imported from the grid which represents a value of %104.2 increase. A similar affect of the link failure can be observed on power exchange RMSE values.

### 3.2. Smart ESS for Link Loss Effects Mitigation

When the link is lost the ESS becomes disconnected from the EMS and therefore is not able to update its setpoints. In this case the ESS can either operate with its last received setpoint or shut down in order to prevent harmful charge/discharge operation. In either of these cases, the optimum MG operation and internal power balancing are no longer maintained.

It is also important to mention that link-losses can happen in different parts of the communication system with dissimilar consequences. For example, if it occurs on the links that transmit the measurements from the inverters to the EMS and the rest of the communication system is operating then false data will be delivered to the ESS. Here, we are interested in investigating total communication failures and other scenarios are out of the scope of this research.

A feasible solution to tackle this problem is to provide and load the ESS with extra information so it can anticipate its upcoming power references. This is compatible to the aforementioned MG structure since all the computations are performed on the external EMS, so in order to obtain the smartness feature in the ESS, load/generation forecastings should be performed on the EMS and extra/redundant information should be transmitted through the communication link.

## 4. Deep Learning Techniques for Load/Generation Forecasting

In this section, the criteria that can be utilized to effectively forecast demand and generation in an MG (residential MG) is presented. As mentioned before, unlike predictable scenarios [6], the randomness and intermittency of both the generation and consumption in MG systems cause difficulties and uncertainties when forecasting their trends. However, noticeable efforts have been conducted in this regard.

In [8], a method based on utilizing artificial neural networks (ANN) for short-term load forecasting (STLF) with a 24-hours time-horizon in an MG scenario was proposed by Hernandez et al. The prediction model of this study consists of two stages where the output of the first stage (maximum and minimum forecasted values for the following day) were used by the second stage that provides the 24-hours prediction data. Wen et al. proposed a recurrent neural network (RNN) algorithm to forecast both the PV-generation and household-consumption in residential MGs for the following day(s). In this study, the uncertainty factor of both the trends were explained and the importance of implementing long short-term memory (LSTM) methodology in the RNN structure was explained [9]. Wind power and PV generation patterns have been forecasted (for the next 24-hours) by using a multistage prediction strategy that was composed of empirical mode decomposition (EMD) and cascade forward neural network (CFNN) algorithms. These predictions were utilized to manage and optimize the consumption from the demand side by providing forecasted generation curves [10]. Kong et al. [31] explained the fundamental differences between forecasting aggregated residential load of multiple users with forecasting a single load. They described the challenges of load forecasting for single residential applications and proposed an LSTM-RNN framework and validate its performance with real-world data.

### 4.1. LSTM-RNN Based Framework for Time-Series Forecasting

It has been proven that RNN methods are a good choice for forecasting time depending data such as load/generation curves for MG systems [32]. An RNN operates in a similar way to other neural networks except it has connections to the previous time-steps. In other words, each cell receives its previous output as an additional input as well as the regular input of the current time step [33]. Although, conventional RNN algorithms perform well for short-term temporal data, extended sequences cause severe delays and memory capacity problems at the learning stage [34].

**Figure 5:**
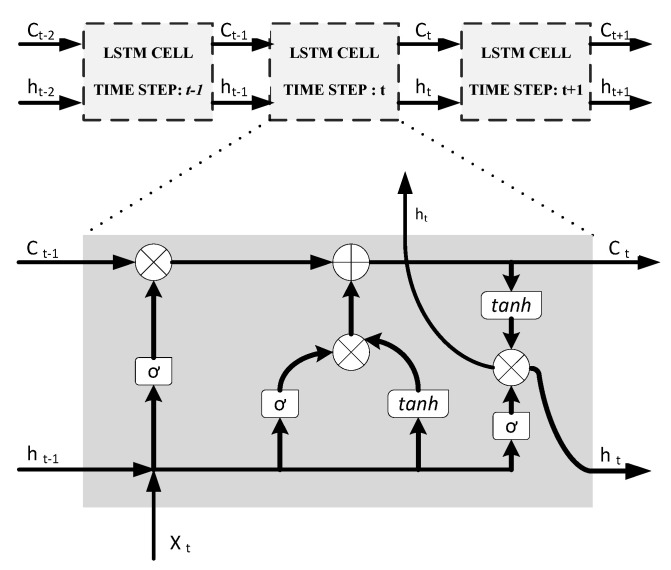
LSTM-RNN cell architecture.

LSTM method was introduced to address these problems and gradually became the dominant RNN method for time-series forecasting [35]. The basic idea of LSTM is that at each time sequence the NN can store and trash some memory data which is performed by using input, output, and forget gates in the algorithm structure [9,35]. An LSTM cell receives the cell state (*C_t−1_*) and the output value from the previous time step which is also called hidden state (*h_t−1_*). In addition, the cell receives a current input for each time step (*x_t_*). The cell output for each timestep are current cell and hidden states, (*C_t_*) and (*h_t_*). It is useful to add, LSTM algorithm utilizes three sigmoid and two tanh non-linear activation functions [35]. An illustration of LSTM architecture is provided in Figure 5.

### 4.2. Simulation Results of Very Short-Term Residential Load Forecasting Using LSTM-RNN

In order to demonstrate the forecasting capabilities of LSTM-RNN a series of tests were performed in this subsection where we attempt to conduct load forecasting, and also in Section 4.3 to repeat the procedure, but this time for PV-generation. The rationale behind these tests is to prove it is possible to predict multiple future time steps of a dataset in the form of a univariate time series by DL techniques and more specifically LSTM. As will be further explained in the rest of the article, the parameters of the DL model should be carefully selected in order to achieve an acceptable prediction while keeping the training time as low as possible.

**Figure 6:**
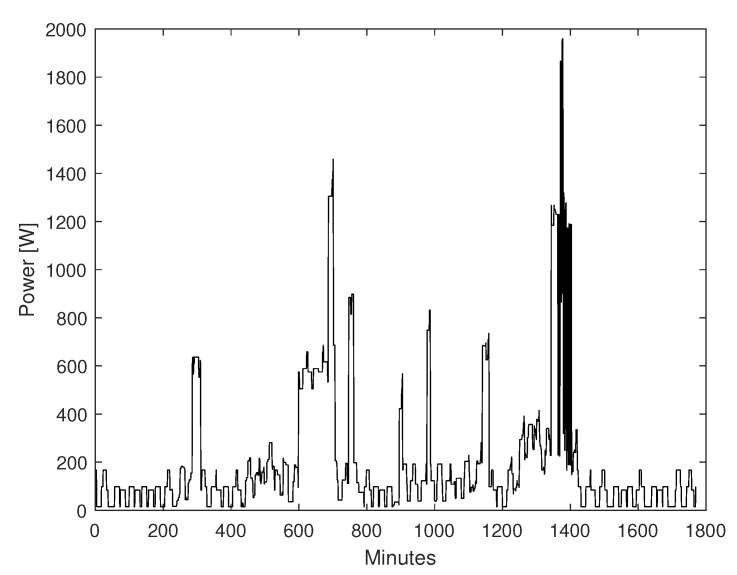
Thirty-hour household consumption power data.

The utilized data for prediction consists of thirty hours of a typical household power consumption generated by utilizing a stochastic model with one minute resolution as illustrated in Figure 6 [36].

The forecasting process is achieved by training the RNN network for 29 h and predict the remaining 60 min. The algorithm was coded in MATLAB programming environment using its standard RNN functions. Different number of hidden units (10, 50, 100, and 300) and epoch # (250, 500, and 2000) were used in order to examine their effects on the RMSE values. Furthermore, two distinct methods were used for forecasting, in the first case, the LSTM-RNN network state is updated with observed (measured) values at each prediction while forecasted data from previous steps were used for the network state update in the second method. The results are summarized in Table 3.

As it can be observed adding the hidden units contributes to lower RMSE values, while extending the training time. However, in case of 300 hidden units the results are worse than the 100 units case; this is due to overfitting of the training set. Furthermore, increasing the epoch number significantly adds to the training elapsed time. This is more noticeable in the case of 300 units when the training time for the 500 epoch # case increases to 25 min and 32 s. Generally, it is safe to state that there is not a clear rule-of-thumb to select the LSTM network parameters and utilizing grid search algorithms can help to optimize the settings.

**Figure 7:**
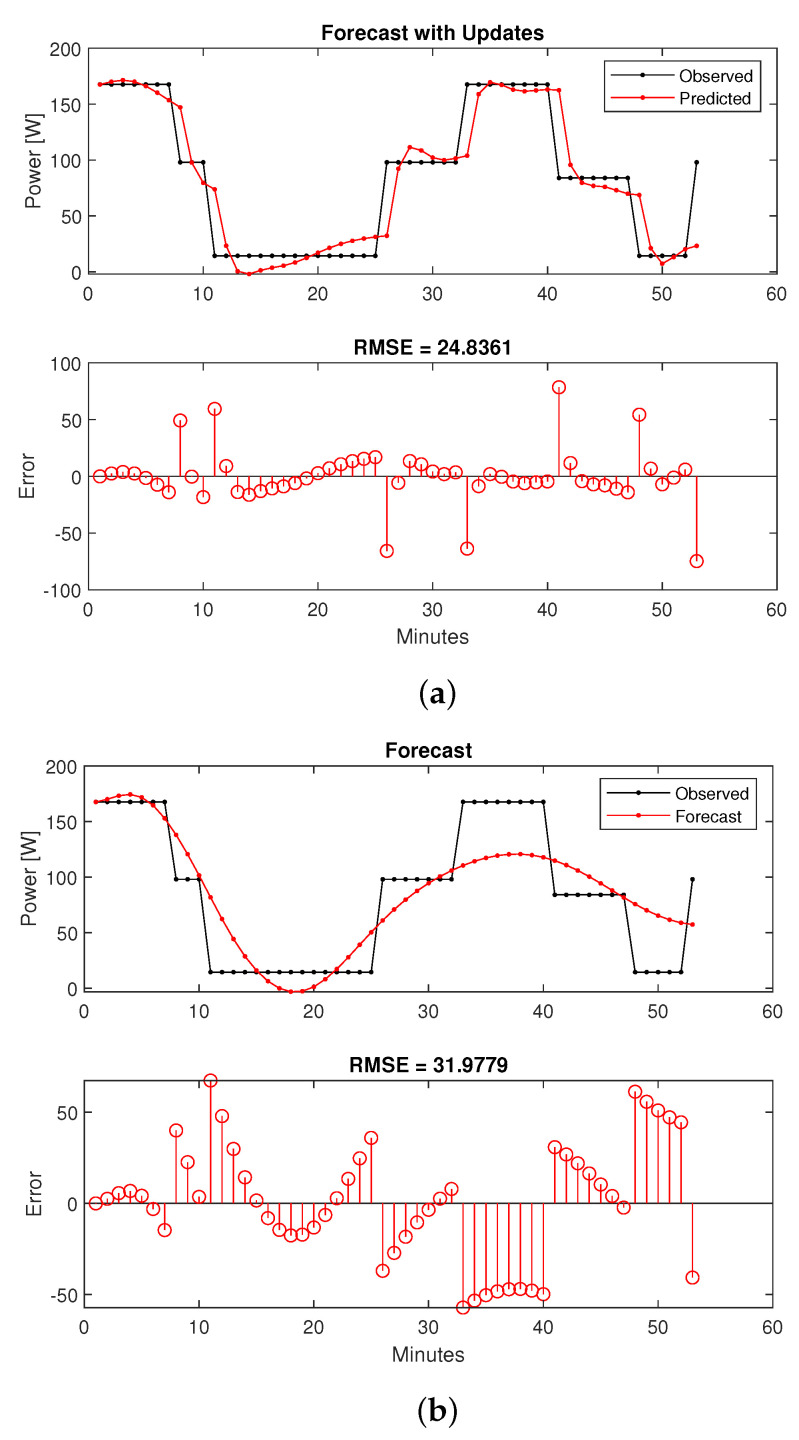
LSTM-RNN very short-term load forecasting: 100 hidden units, 500 epoch #. (**a**) Updated with observed values. (**b**) Updated with predicted values.

Based on the test results a satisfactory configuration of 100 hidden units and 500 for epoch # was chose. The two distinct sets of forecasted results are illustrated in Figure 7. As expected, the prediction accuracy is much higher in Figure 7a (with updates) in comparison with Figure 7b. However, a decent approximation is still available from the second method. In our application, the observed values are not available since our goal is to provide load/generation predictions for several future time-steps so the EMS can calculate and transmit extra power references to the ESS. In other words Figure 7b represents the forecasting scenario that will be used for our proposed framework.

### 4.3. Simulation Results of Short-Term PV Power Forecasting Using LSTM-RNN

Following the same prediction criteria as before, in this section, the LSTM-RNN framework is used for short-term PV-power forecasting. However, the selected training period and forecasting time-horizon are different from the previous case. This is due to the characteristics of solar power that follow a daily-base pattern and depend on geographical and seasonal parameters. This is in contradiction with household load trends that follow appliances scheduled consumption or arbitrary user decisions.

**Figure 8:**
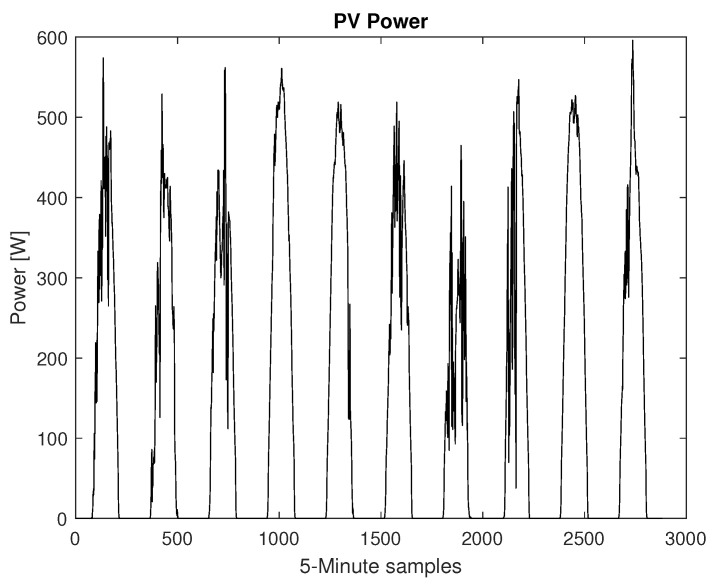
Ten days of PV-power dataset.

A database of synthetically generated PV-power measures provided by the National Renewable Energy Laboratory (NREL) of the U.S. Department of Energy was used for this simulation [37]. From the database ten days (1 January 2006 to 10 January 2006) of solar generation data corresponding to the state of Alabama were selected with 5 min resolution, Figure 8. The LSTM-RNN was trained for 9 days and predicted PV-power for the following day. It should be noted that solar generation strongly depends on immediate weather conditions and phenomenon which was not considered in this simulation and more complicated algorithms are essential for very-accurate predictions.

**Figure 9:**
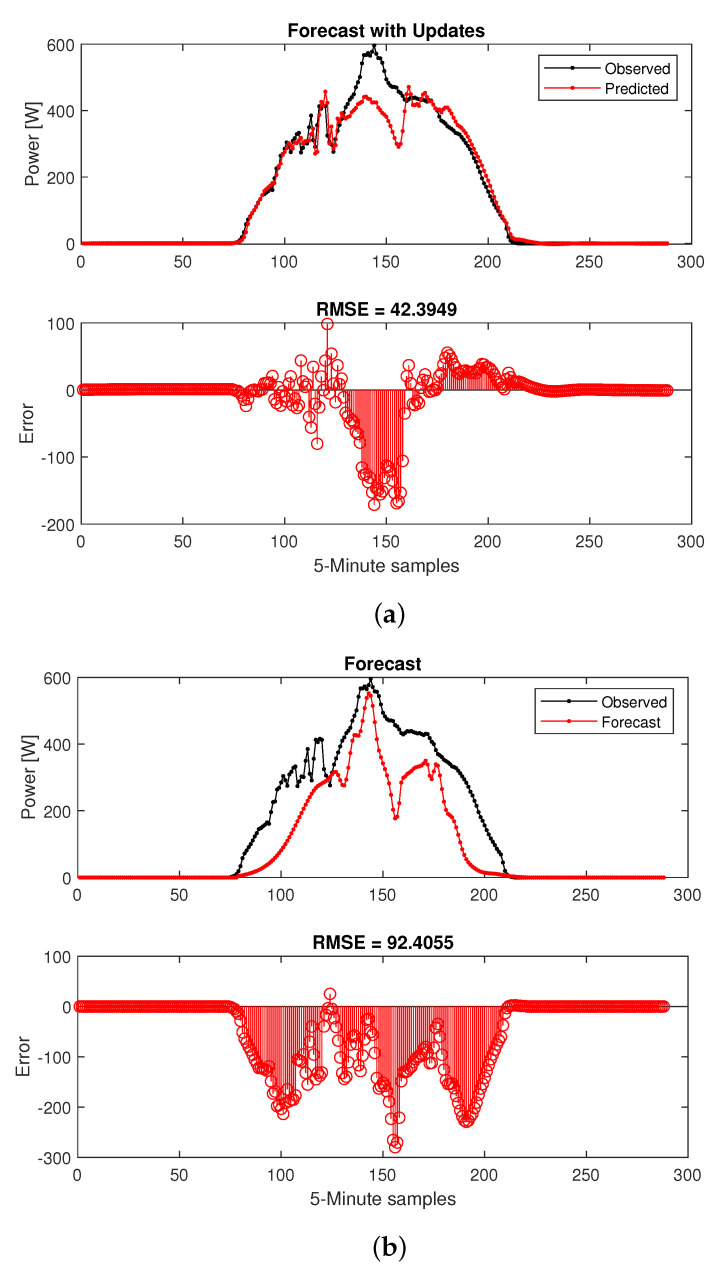
LSTM-RNN short-term PV-generation forecasting: 200 hidden units, 1000 epoch #. (**a**) Updated with observed values. (**b**) Updated with predicted values.

The results are presented in Figure 9, where 200 hidden units and 1000 epoch # were selected as the LSTM-RNN prediction settings. Two different trends are provided that correspond to the two cases of network update with observed values and forecasted values from previous time steps. Similar to the load forecasting test, when the networks is updated with observed values, Figure 9a, the prediction accuracy is much higher, however, this criteria is not suitable for the application of our interest as discussed in the previous section.

It is obvious that by using different LSTM-RNN parameters settings (other than the ones of these tests) various levels of prediction accuracy can be achievable. However, performing further tests to produce better forecasting results is out of the scope of this paper since here we are presenting the practicality of performing sensible predictions on time dependent data (Load/PV-Generation) by using LSTM-RNN algorithms as a proof of concept.

## 5. Communication Protocol and Latency

In this section, a short description of the communication messaging protocol that is used in our MG testbed configuration accompanied by latency analysis and detailed discussions have been provided.

### 5.1. Messaging Protocol

There are different application layer protocols that can be utilized for such applications. The more famous ones are hypertext transfer protocol (HTTP), message queuing telemetry transport (MQTT), and constrained application protocol (CoAP) [38]. MQTT, which is the selected messaging protocol in our aforementioned MG architecture, is considered to be a better candidate for device-to-device (D2D) applications due to its publish/subscribe methodology and simpler frame structuring which means smaller headers sizes than the HTTP protocol [39]. This provides the light-weight feature of MQTT. It is necessary to mention that in our proposed structure IEEE 802.11 (WiFi) is utilized as the physical layer protocol.

MQTT frames consist of a fixed header (minimum one and maximum two bytes), a variable header and payload that can theoretically be as large as 260 MB. The formatting of MQTT messages are comprehensively explained in reference [39]. It is important to mention that, while binary messages are also possible, normally the payload bytes are formed by UTF-8 encoded strings in MQTT protocol. In other words, if the messages are going to grow in size due to adding supplementary information “*at least one byte*” will be added to the payload for each character. The effects of this phenomenon is investigated in terms of latency increase, due to larger frames, and possible medium access control (MAC) layer collisions in the next subsection.

Since the idea of this article is related to transmission link failures it is important to provide a brief discussion about the failure rate or the reliability of the utilized communication framework. This is required to be discussed from two different points of view which are MQTT reliability as the messaging protocol and the rest of the transmission architecture that is conceptualized as the OSI stack:

- The reliability of the MQTT messaging protocol depends on the level of quality of service (QoS) selected. Generally, a larger level of QoS translates to higher reliability and QoS 1 and QoS 2 ensure the delivery of messages while increasing the latency as a consequence of adding the number of transmissions and redundancy [2].
- MQTT works on top of TCP/IP and a compatible physical layer protocol, which in this case is WiFi. The failure rate of this combination is totally case sensitive and depends on lots of factors such as the size of the network, congestion issues, security level, and power availability/stability [40].

Based on the points addressed above, it can be argued that failure rates or reliability of a communication architecture such as MQTT depend on different factors regarding the specific application. However, it can be stated that using higher MQTT QoS levels removes packet loss possibilities from the application layer. Then, further down the OSI stack, employing a stable and standard network such as WiFi can greatly contribute to higher reliability.

In the case of the tests conducted for this study, as expected, no unintentional packet losses or link failures were observed since MQTT QoS 1 was employed, limited users were connected to the LAN, and also the very robust and high bandwidth IEEE 802.11ac was employed for the link and physical layer. All the failures, that will be introduced in the following sections were intentional to emulate poor network scenarios.

### 5.2. Latency Experiment with Various Payload Sizes

There are several elements that contribute to the overall end-to-end latency in a wireless communication system. They are processing/queuing latencies, MAC latencies and transmission latencies [41]. Among those, the transmission latencies depend on the size of the header(s) and payload of a specific communication frame. The latency measurements in this study are only focused on the effect of the size of the payload on the overall MQTT latency, more comprehensive MQTT latency measurements and analysis, and also including the effect of different MQTT QoS levels can be found in [2]. This is because it is required to work out what will happen when an enhanced message is being sent from the EMS that can be considerably larger than a normal message with a single payload, and finally to determine if the time on air (ToA) of the enhanced message is acceptable. Needless to say, if the enhanced message latencies become too large then the whole communication system will no longer be suitable for this particular application.

**Figure 10:**
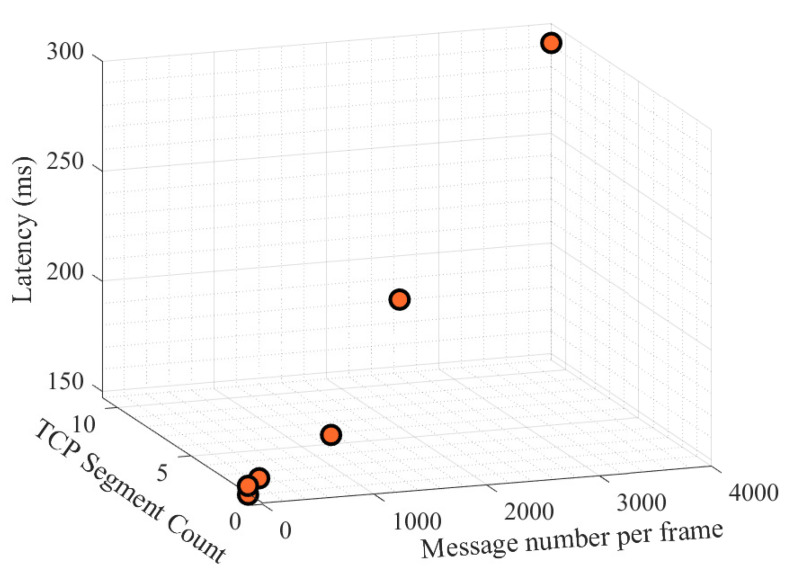
MQTT end-to-end latency results. The effect of TCP segmentation is clearly visible on the different measured latency values.

An experimental testbed consisting of two PCs, where MQTT clients were created using python-paho package, and a mosquitto MQTT broker was implemented to evaluate the effect of changing payload size on end-to end MQTT latency. In this test, different payloads consisting of 1, 10, 100, 1000, 2000, and 4000 numerical values (three digits) that each of them can represent measurements or references were tested. By comparing the transmit and receive timestamps, latency for a single transmission is calculated. An average value of ten iterations per each payload size produced a realistic estimation of overall latency for different cases. The results are presented in Table 4 and Figure 10.

It can be observed from the results that for the cases of less than 1000 messages the latencies remain fairly constant with a value of approximately 150 milliseconds. Beyond this point, the latencies increase since the payloads need to be divided to a number of TCP segments. It should be noted that the maximum size of a TCP segment is limited to 1500 bytes including its IP and TCP headers [42]. The payload sizes and TCP segment counts of the MQTT frames are obtained by using Wireshark which is an open source and widely recognized network protocol analyzer.

The following are key notes and findings regarding this experiment:

- In order to measure the latencies the transmitter and receiver clocks should be synchronized. Since two separate PCs were utilized, using windows internet-time and synchronizing them to the same time-server seems to be a feasible option. However, in practice, this method did not provide sufficient synchronization accuracy, especially when measuring latency in the millisecond scale. Therefore, we synchronized our transition and reception timestamps to a network time protocol (NTP) server, specifically “*pool.ntp.org*”, in the python programming environment.
- The fact that latency values are relatively unaffected for payloads containing less than 1000 numeric values is not unexpected. This is because the testbed WLAN physical protocol is IEEE 802.11n with a nominal link speed of 45 (Mbps). Comparing the scale of this bandwidth with the size of the experimental messages payloads proves the aforementioned fact.
- The measured value of 150 milliseconds that was recorded for most of the packets was not related to transmission latencies (due to the tiny size of frames and abundant available bandwidth of the channel). Furthermore MAC latencies were also not present, therefore this shared value only represents processing and queuing latencies.
- For the previously explained application of this research, which the communication system is designed to be used, the redundant information is planned to be in type of power references for several future time steps. That means a number of added numerical values in the scale of 50 or less, therefore the payload sizes will not exceed the upper limit of this experiment.

## 6. Proposed EMS and ESS Architecture

**Figure 11:**
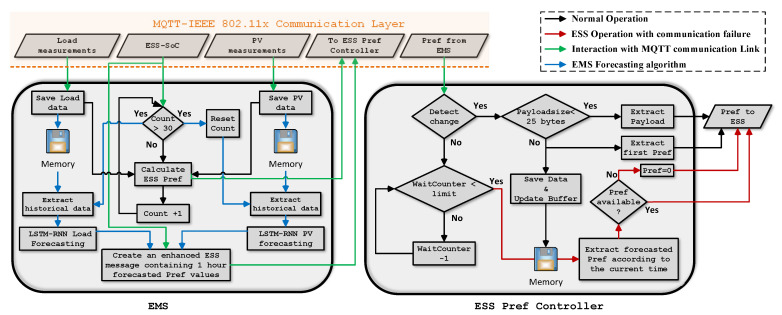
EMS and ESS Pref Controller architecture to transmit and receive enhanced power references with redundant information.

Following the discussions in the previous sections regarding load and PV-generation short-term forecastings, in this section the proposed structure of the EMS and also the ESS power-reference controller is presented. Figure 11 illustrates the aforementioned architecture.

As it can be observed, the EMS receives load and PV-generation generation values from the smart-house sensors through the MQTT communication system. The ESS-SoC is also transmitted to the EMS by the same link. Depending on the particular time-step, the EMS either calculates the immediate power reference for the ESS or perform load/PV-generation forecastings and prepare an enhanced message that contain ESS power references for multiple future time-steps. In this proposed architecture, assuming that the measurements sampling rate is 1 minute, EMS forecastings are performed every 30 min with 1-hour time horizon so that the ESS has forecasted setpoints for at least 30 min in case a communication failure occurs immediately before a scheduled EMS forecasting.

**Figure 12:**
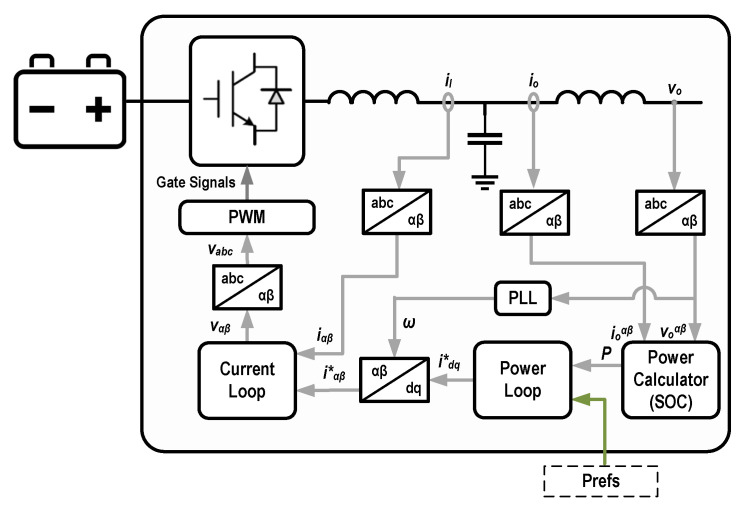
Control structure of grid-feeding converters.

Once these power-references are transmitted from the EMS, they will be received by the ESS while it is subscribing to the same topic in the MQTT communication infrastructure. In MG methodology, ESSs (similar to DGs) are controlled by power-electronics based converters. The experimental testbed configuration used for this research that was explained in Section 3.1 is based on grid-feeding inverters operating in grid-connected mode. In such a configuration, the transmitted power references are required to be consumed by the converter’s controller power loop to regulate the power exchange with the MG bus. This scheme is illustrated in Figure 12.

The EMS power-references are directly utilized to generate pulse width modulation signals that coordinate the charge and discharge operations of the ESS. However, prior to this step, there should be another controller that investigates the operational conditions of the communication link and select what type of data (real time or forecasted) should be provided to the converter’s main controller. This is described in the right hand side block (ESS Pref Controller) of Figure 11. As it can be observed, the link health status is first checked by performing a wait loop. If no change is detected in the received Pref (from the EMS) for more than one minute the controller assumes a link-loss has occurred and attempts to use the forecasted values stored in its memory storage. These predicted Prefs were previously stored by recognizing an “*enhanced EMS message*” (according to its larger size) from a normal message. The main characteristics of the proposed EMS-ESS structure are listed bellow:

- The measurements and also EMS generated Prefs rates are every 1 min.
- The load and PV-generation forecasting’s time-horizon is one hour with 5 min resolution.
- Enhanced messages with redundant (predicted) Prefs are created every 30 min.
- Based on the experiments presented in Section 5.2 a normal message contains approximately 20 bytes while enhanced messages can be between 200 and 300 bytes in size. It is important to mention that, the byte # uncertainties are due to the possible different number of digits of Prefs. However, It has been proven earlier that the end-to-end latencies do not significantly change when and increase in payload size of this scale occurs.
- When using saved data, if the Pref controller cannot find a stored Pref for a specific instant, the value of zero is send to the ESS controller as power-reference. This happens when the communication outage duration prolongs and all the saved values are used.

### 6.1. The Benefit of the Utilization of Forecast Data in the Case of a Transmission Link Loss

This study started with the conceptualization of MG communication systems, followed by demonstrating link loss effects on the performance of a typical grid-connected MG in terms of PCC power exchange. After that, a possible solution was proposed that was equipping the EMS with predicted values of consumption and generation. It was demonstrated how these predictions can be achieved by using DL techniques and after that, by transmission latency analysis it was proven that enhanced messages with a larger payload will not considerably affect the latency. Finally, a proposed EMS and ESS structure was proposed to create and manage enhanced messages to mitigate link-loss failures earlier in Section 6.

In this part, the attempt is to show by using the proposed architecture how the effects of transmission link-loss can be mitigated in a quantifiable manner. For this purpose, the short term load/ PV-generation forecasting results from Section 4.2 and Section 4.3 were employed and a comparison of MG power performance in normal and faulty (communication link-losses) situations is conducted. In other words, the power exchange pattern with the grid at the PCC is analyzed when the ESS is operating with accurate EMS power-references originated from real-time measurements or when the EMS calculations are based on forecasted values.

In order to achieve this goal, one-hour of load and PV-generation values with 5-min resolution of the dataset used for experiments in Section 3.1 is selected and the differences between consumption and generation values of the same time steps are calculated. By this the internal power imbalance can be worked out. This process has also been conducted for the forecasted values of the same time steps that are calculated by the methods explained in Section 4.2 and Section 4.3. The aforementioned calculated values represent the ESS power references assuming that the instantaneous ESS-SoC value permits for a valid charge/discharge operation.

**Figure 13:**
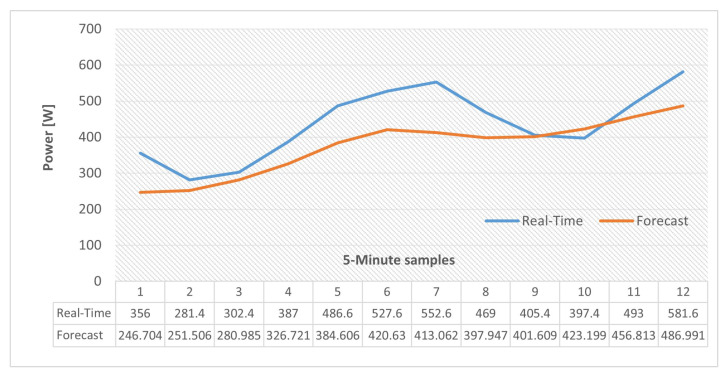
Internal power excess/deficit inside the MG (One hour test).

The results are provided in Figure 13. In this selected time horizon, since the PV-generation values are consistently larger than the consumption there is power excess inside the MG which is represented by the black curve in the figure. On the other hand, it can be observed that the forecast curve follows the pattern of the actual values in an acceptable manner. For this time period the total internal energy imbalance inside the MG is (0.4367 kWh) which means if an operational ESS is present in the system, (0.4367 kWh) energy will be stored while using accurate Prefs. This is only possible if an operational communication link between the EMS and ESS exists.

Needless to say, if forecasted values are used in the case of communication link loss, the ESS cannot compensate fully for the internal power imbalance at every time-interval and power exchanges with the main grid are necessary. In the case of this test, using the forecast values by the ESS results to the storage of (0.3742 kWh) of the aforementioned energy value, which means even if some is lost (injected into the grid) still the result is much better compared to a situation where all of it is exported in the case of an internal imbalance when the transmission link is down. This is the situation indicated by red arrows in our aforementioned architecture, Figure 11.

**Figure 14:**
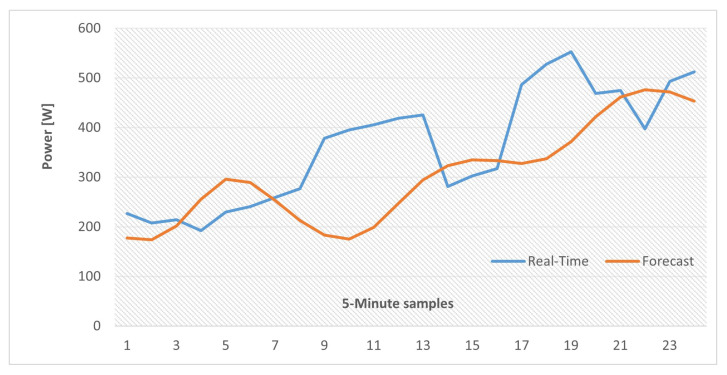
Internal power excess/deficit inside the MG (Two hour test).

**Figure 15:**
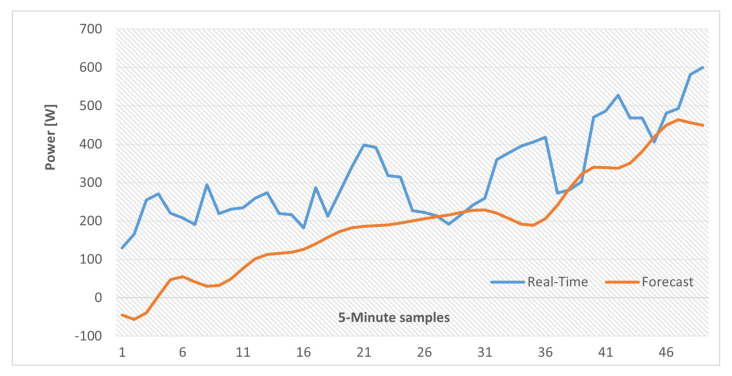
Internal power excess/deficit inside the MG (Four hour test).

In addition, similar results are also provided with actual and forecast data for two and four time periods and the results are illustrated in Figure 14 and Figure 15. It should be noted that, in the case of these tests, the LSTM-RNN network state was updated by using forecasted values at each time step (as explained in Section 4.2) so errors will accumulate when the time horizon is expanding. To compare these scenarios, the amount of energy imbalance based on actual load and PV-generation values, that would be the real-time EMS power references, in the case of an operational communication link, along with the same references calculated by using forecast values are gathered in Table 5.

As it can be observed, although by expanding the time window the energy exchange with the grid increases, utilizing the forecasted values is still beneficial. Otherwise, the ESS must be shut down after a link failure, since references are no longer received, and all the MG internal energy imbalances which are represented by the values of the first row of Table 5 should be compensated by the grid.

### 6.2. System Performance Comparison for Different Link-Loss Occurring Instant

Based on the proposed EMS-ESS system explained at the beginning of Section 6, it can be noted that there is a particular architecture for transmitting enhanced messages (every thirty minutes with forecast data for the next hour) so it is crucial to analyze the system’s performance for different faulty scenarios. The difference between the analysis of this subsection with Section 6.1 is that, here, it is attempted to monitor the system performance regarding the occurrence time of the fault, while in the former analysis it was assumed that the ESS is loaded with forecasted setpoints for different time-windows.

**Figure 16:**
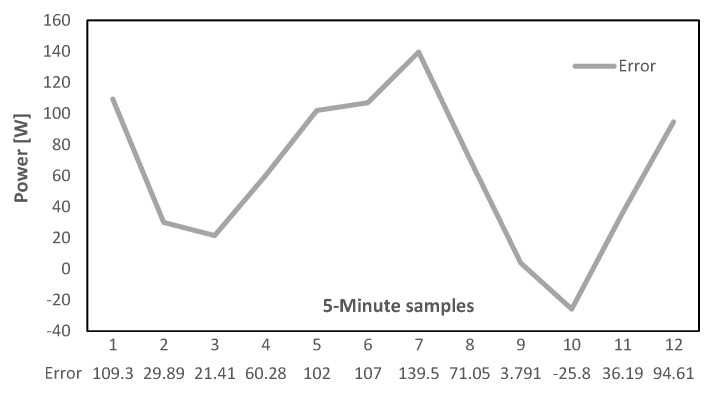
Error due to utilizing forecasted values, first case.

Two extreme cases are considered in this part, in the first case it is assumed that the communication link is lost for a whole hour, and this loss occurs immediately after an enhanced message was transmitted, therefore, the ESS has the necessary Prefs for the next hour. Due to the availability of forecasted Prefs for the duration of the fault, the ESS can properly operate. However, errors exist since forecasted values never exactly match with the real measurements. Figure 16 illustrates this error at each time step which will accumulate to a total of (0.0625 kWh) of electrical energy injected to the grid at the duration of this communication link loss. This can also be represented in terms of PCC power exchange RMSE (0.0273 kW) which indicates a significant improvement to the link-loss situation with no stored ESS power setpoints where the RMSE of the same trend is as high as (0.4464 kW).

**Figure 17:**
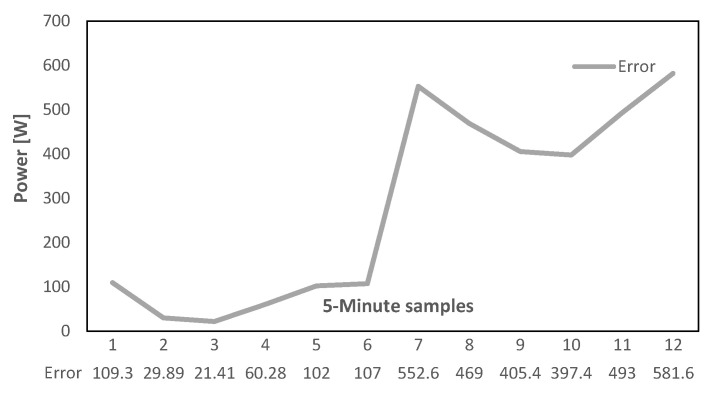
Error due to utilizing forecasted values, second case.

In the second fault scenario, the link loss happens just before the scheduled enhanced message is generated and transmitted from the EMS. Like the previous case, this fault also lasts for an hour. Regarding the circumstances, the Pref controller memory buffer only contains forecasted power references for the next 30 min and after that the setpoint will be changed to zero since there are no more data available. The error between ESS power references and the proper values that would have been generated and transmitted by the EMS with an operational link is illustrated in Figure 17. As it can be observed there is a sudden steep increase after 30 min, which is due to setting Pref values to zero from this point forward. During this fault, the total amount of (0.2774 kWh) of electrical energy is injected to the grid and the PCC power exchange RMSE is (0.3497 kW) which both are considerably greater than the previous scenario where the forecasted values were available for the whole communication outage period. However, the performance is still better than the base scenario where no mitigation technique was implemented.

**Figure 18:**
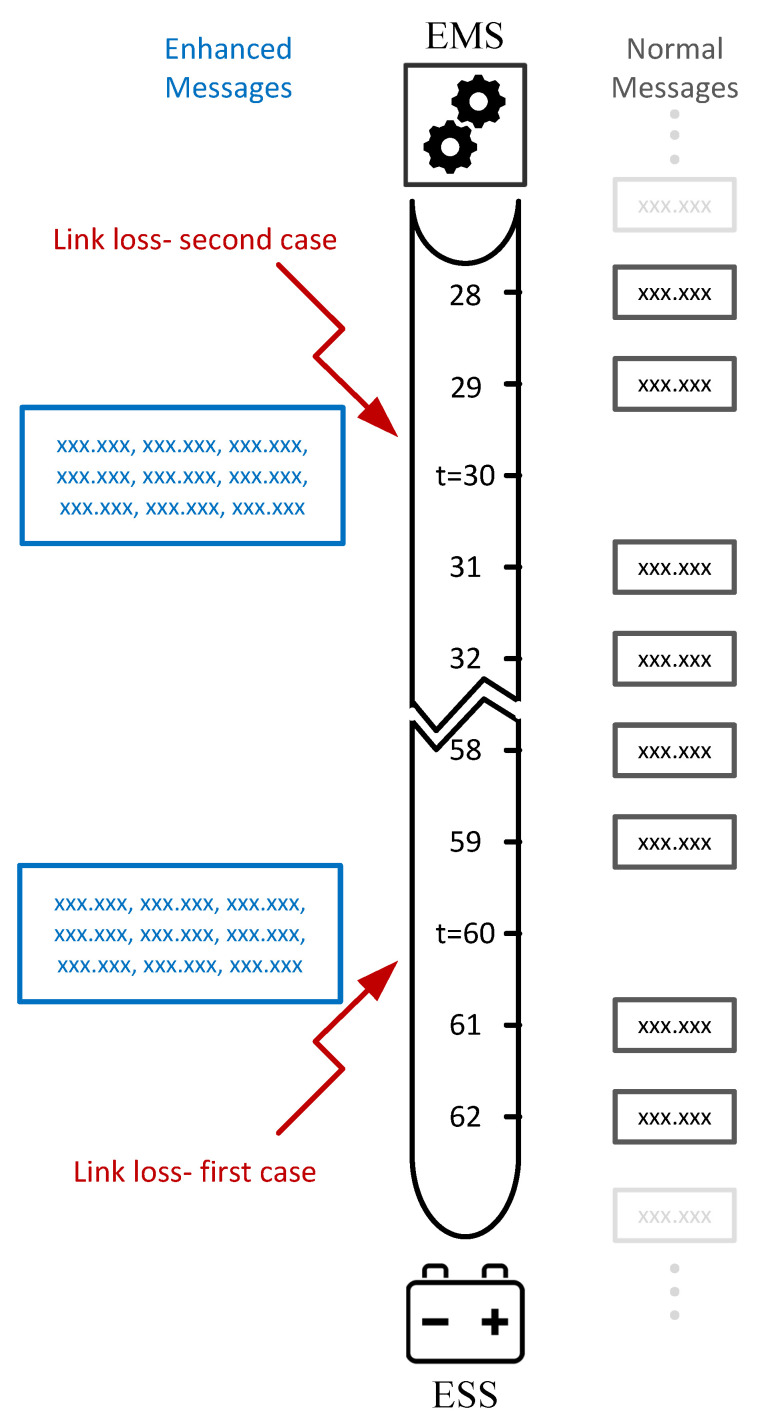
Two different cases of link losses.

The two link-loss scenarios discussed above are illustratively explained in Figure 18.

## 7. Discussions and Conclusions

In this paper, we propose a scheme to mitigate the problems caused by communication system malfunctions on MG control and management. Our work combines three different, but related, areas of research, which are MG power management, M2M communication, and DL based energy consumption and production forecastings.

Previous studies that focus on load/generation forecastings for MG control and management did not deeply focus on communication aspects. On the other hand, it is challenging to find a communication-related MG publication that include DL based load/generation forecasting. That is to say, the authors could not find a similar study in the current literature that equally covers all the considered aspects of this work. Furthermore, the concept of enhancing the communication message content that transmits power references from an external EMS to an ESS in a typical grid-connected MG configuration has novelty and justifies that this proposal can empower the existing literature.

For the sake of providing a definite discussion on the findings of this research several points can be noted as follows:

- The effects of payload sizes on end-to-end communication latencies were experimentally tested. The results proved that by enlarging the messages in the scale required for this application no serious latency increases were observed.
- It was proposed to use RNN-LSTM algorithms for load and PV generation forecastings and simulation results proved that although the forecastings were not always perfect but the general patterns were consistent.
- The unfavorable energy interactions with the grid through the PCC in case of communication link failure was analyzed. It was shown that these deviations from internal energy balancing can be mitigated by designing a more capable communication infrastructure that can prepare, arrange, and transmit enhanced messages with forecasted power references for next time steps. This helps the ESS to anticipate its upcoming, however, not 100% accurate setpoint in the case of communication link failure.
- Numerical evaluation for internal energy balance of the MG by using the proposed communication architecture and utilizing the LSTM-RNN load/generation forecasted values was conducted and the results were presented for two different communication link loss scenarios.

In summary, the findings of this paper demonstrate how some detrimental effects of communication outage on the optimum operation of MGs can be minimized by enhancing message’s content with the help of utilizing deep-learning methods. This is not a simple and trivial problem, therefore, future work will be required so that the concept can be further investigated. Potential future research topics will be focused on considering different MG configurations such as islanded MGs based on VSIs and also other communication protocols and infrastructures. In addition, other deep-learning based forecasting methods will be investigated and their capabilities to perform short-term or very short-term predictions will be evaluated. The aforementioned future works are just some examples and the potential of expanding the concept introduced in this paper is vast according to its originality.

## Figures and Tables

**Table 1:** Different network levels, devices, communication requirements, and protocols.

Network	Level	Node Types	Characteristics	Protocols	References
**DAN**	*Device*	Converters, V/I Sensors, Data Loggers, Data Acquisition	Low-Range, High-Speed, Ultra-reliability	Can-Bus, Modbus, Interbus, Profibus, Ethernet	[19,20,21]
**SHAN**	*Smart Home*	Smart Applications, Control, Monitoring, Processing	Heterogeneity, Scalability, Mobility	ZigBee, WiFi, Zwave, Bluetooth, 6LoWPAN	[14,22,23,24]
**MAN**	*Microgrid*	DGs, ESSs, EV Charging	Long-Range, Independent, Security	LoRaWAN, SigFox, Dash7, Ingenu, NB-IoT	[25,26,27]
**MMAN**	*Multi- Microgrid*	Energy Internet	Ultra Long-Range, Terrain affected, Standardization	4G, 5G, Direct to satellite LPWAN	[28]

**Table 2:** Comparison between base scenario and MQTT-based EMS.

	PV	Load	PCC No Control	PCC EMS	%Change
Energy (kWh)	2.145	−2.599	−0.455	0.019	−104.2
Power RMSE (kW)	-	-	0.191	0.029	−96.8

**Table 3:** Load forecasting RMSE values for different LSTM network hidden units and epoch #.

Hidden Units	Epoch #	Training RMSE	RMSE on Test Set (with Updates)	RMSE on Test Set	Training Time
10	250	0.36	27.9511	91.4892	0:30
10	500	0.36	27.9075	83.9501	0:54
10	2000	0.36	27.9057	83.3967	3:22
50	250	0.23	26.7577	51.7804	0:49
50	500	0.21	26.7284	58.8833	1:33
50	2000	0.21	26.6763	58.2284	6:26
100	250	0.20	24.771	31.3054	2:00
100	500	0.18	24.8361	31.9779	3:37
100	2000	0.18	24.8239	31.4652	14:12
300	250	0.20	27.9456	58.9637	12:56
300	500	0.14	27.6778	199.3257	25:32

**Table 4:** Latency test results for various payload sizes.

Number of Data	1	10	100	1000	2000	4000
Latency (milliseconds)	147	151	154	161	205	291
Payload size (bytes)	21	89	418	4017	8016	16,018
TCP Segment count	1	1	1	3	6	11

**Table 5:** Comparison between the PCC energy exchange for different scenarios.

	One Hour	Two Hours	Three Hours
ESS energy (charge/discharge)			
while receiving real-time data	0.4367 kWh	0.7239 kWh	1.240 kWh
ESS energy (charge/discharge)			
working on forecast data	0.3742 kWh	0.6059 kWh	0.7196 kWh
Energy exchanged with			
the grid (Export/import)	0.0625 kWh	0.1180 kWh	0.5204 kWh

## Data Availability

Not applicable.
